# Effect of Stone Impacts on Various Ground Engaging Tools (Flexible/Stiff Tines and Coulter): Part I

**DOI:** 10.3390/ma15041568

**Published:** 2022-02-19

**Authors:** Aleksander Lisowski, Adam Świętochowski, Magdalena Dąbrowska, Jacek Klonowski, Tomasz Nowakowski, Jarosław Chlebowski, Przemysław Tryskuć, Tomasz Parys, Samuel Ferré, Martin Roberge

**Affiliations:** 1 Department of Biosystems Engineering, Institute of Mechanical Engineering, Warsaw University of Life Sciences, Nowoursynowska 166, 02-787 Warsaw, Poland; aleksander_lisowski@sggw.edu.pl (A.L.); adam_swietochowski@sggw.edu.pl (A.Ś.); jacek_klonowski@sggw.edu.pl (J.K.); tomasz_nowakowski@sggw.edu.pl (T.N.); jaroslaw_chlebowski@sggw.edu.pl (J.C.); przemyslaw.tryskuc@gmail.com (P.T.); tomaszparys11@wp.pl (T.P.); 2 Soil & Crop Modelling Team, CNH Industrial Canada Ltd., 1000 71st Street East, Saskatoon, SK S7P 0A3, Canada; samuel.ferre@cnhind.com (S.F.); martin.roberge@cnhind.com (M.R.); 3 Chemical & Biological Engineering Department, University of Saskatchewan, 3B48 Engineering Building, 57 Campus Drive, Saskatoon, SK S7N 5A9, Canada

**Keywords:** mechanical engineering, stone sphericity, stone movement, stone throwing distance, specific work

## Abstract

Analysis of the state of knowledge showed a gap in the description of tool–stone feedback. Therefore, the aim of this study was to investigate tool–stone interactions. Spherical-like silicate stones were hit by stiff and flexible tines with a duckfoot or a coulter. The tools worked with various parameters in the depth range of 0.05–0.10 m and a speed of 0.83–2.22 m·s^–1^. The characteristics of stone movement were specific to the type of tool and were described by the Numerical Stone Movement Scale developed for the purpose of the research. After the impact with the stiff tine, the stones were thrown the greatest distance of 0.26–1.08 m, and these distances were strongly dependent on the working speed and slightly dependent on the working depth. Large vibrations of the flexible tine and the location of the contact point of the tine in relation to the centre of the stone thickness contributed to the random behaviour of stones that were slightly moved, rotated or displaced. The specific work required to remove the stone reflected the distance travelled by the stone as well as the specific force which largely contributed to increasing the differences in this work between both tines.

## 1. Introduction

Agricultural implements for soil cultivation—disc harrows, stubble cultivators, hillers, and weeders—are tools towed by tractors and do not have advanced electromechanical elements providing protection against hitting an obstacle, e.g., stones in the soil [1]. Stony soils create serious problems in the work of tools and working elements. Large stones, especially hard flints, can bend or break the working parts of passive or active hoes. Complex construction hoes can be equipped with various types of working elements, e.g., knives, duckfoots, and chisels. The working elements can be fastened with shanks and flexible or stiff tines [2]. 

Working elements mounted on flexible tines favor soil aeration, destroy weeds, e.g., by tearing up troublesome couch grass, loosen caked soil and cover the applied fertilizers. The main task of the duckfoots attached to the stiff tines in the cultivators is to trim weed roots. Disc coulters of various diameters, profiles and cutting edges are also used in cultivating tools. The construction of compact disc harrows with individual disc suspension allows them to be protected against stones [3]. Designing cultivation tools that will be able to work in stony soil is a challenge for scientists and manufacturers of agricultural machinery. Materials resistant to abrasion, thermo-chemical treatments increasing resistance to variable loads, and shapes with variable geometry both for tines or shafts and working elements are used. Flexible tines of the S-type have good features allowing the use of the highest working speed. Flexible tines work well on most harvest residues without clogging and are not easily damaged by stones. On the other hand, stiff tines ensure good work on hard and stony soil and with a strong infestation of quack grass and other weeds with underground rhizomes [4]. In order to reduce mechanical damage to the elements working in stony soil, their design is focused on strength properties; reliability; ease of maintenance and repair; and cost-effectiveness [1].

The improvement of tools is achieved by conducting research on innovative solutions of single knives [5], complete tools [6] and theoretical analyses [7]. Theoretical models are the most valuable for science, but they are mainly based on neural network applications and finite element and discrete element methods, and do not take into account elastic stresses in the variable geometry of working elements [8]. The variability of the geometry of working elements also makes it difficult to infer qualities from the results of experimental tests, because it is necessary to take into account the instantaneous dynamic values of the measured parameter and correlate it in time with variable shear parameters [9]. Correlation of the output signals is possible for single working elements. It was found that increasing the value of tine elasticity initially causes an increase then a decrease in the value of vertical force and penetration, regardless of the shear rate, which is described by the second-degree parabola equation. An increase in the cutting depth causes a linear increase in the vertical force, while the force gradient decreases with increasing tine elasticity [8]. The tractive force and soil disturbance are influenced more by the depth of the working tool than the working speed [10]. On the other hand, there are no results of the research on the behavior of the tine or the cutting element during impact with a stone embedded in the soil.

Self-excited vibrations are generated in narrow flexible tines during soil loosening. These vibrations depend on the elasticity of the tine and the variability of the soil resistance [11]. The cross-sectional area of the cut soil changes under the influence of the amplitude and frequency of vibrations, which means that through vibrations the quality of loosening is higher. As the oscillation of the tool increases, the vertical and horizontal forces of the working elements coming from the soil decreases [12]. There is no information as to whether or not the resulting oscillations of the spring tools can affect the displacement of the stones and whether interactions that will change the behavior of the tool or stone can arise.

The stiff tines of cultivators and subsoilers are highly susceptible to damage from stone contact. For this reason, various types of protection for stiff tines are commonly used, such as a shear pin, flat springs and hydraulic cylinders with a gas-hydraulic accumulator. In order to design wear-resistant tools and effective protection against shock overload caused by stones or other objects, it is necessary to study stone–tool interactions with particular emphasis on initial contact conditions.

The evaluation of tools working in the soil is based on maximizing the volume of loosened soil with the lowest possible cutting forces [13]. Under typical soil conditions, soil–tool interactions are characterized by the 3D forces at the soil–tool interface and the soil disturbance [14]. The loosening effect in the form of an increased volume of soil pores in the disturbed zone depends on the stresses and the initial condition of the soil [15]. 

There are very few articles in the available literature [16] that present the results of research on the movement of stone embedded in non-cohesive soil disturbed by a moving tine. The stone was represented by a set of three, mild-steel hemispheres of different diameters. It was found that the hemispheres do not move until their centers exceed the limit of soil destruction in front of the tines. It was also found that sand did not always cause the movement of the hemisphere before contact with the tine; this movement was determined by the location of the center of the hemisphere in relation to the soil movement limit in front of the tine. This effect was independent of the speed. A minimum hemisphere size of 20 mm was found, below which the movement always started before contact with the tine. Damage to the cutting edges of agricultural tools can be reduced by designing the tool to create a large area of damaged soil in front of the cutting edge. These research results are interesting, but cannot be directly put into practice due to the idealized conditions of the research. The authors [16] pointed to the limitations and suggested investigating the behavior of an object with less regular shapes than a ground mild steel hemisphere. This conclusion acted as another inspiration to undertake our own research with natural stones.

The aim of this study was as follows: (1) to investigate the ground engaging tool–stone relationship during an impact with a duckfoot mounted on a flexible or stiff tine and with a coulter—elements working in the soil at different depth and working speed; (2) determination of the distance travelled by the stone after the tool collides with the stone; (3) calculation of the slope factor of the straight line and the angle of stone movement relative to the direction of the tool movement; (4) development of a new indicator: the Numerical Stone Movement Scale (NSMS); and (5) calculation of the directional coefficient of the specific force, resultant in the horizontal plane and the specific work required to remove the stone embedded in compacted soil.

In order to reduce random factors, the studies were carried out in the controlled conditions of a soil bin facility.

The novelty of the article is to present for the first time the original test method and describe the behavior of the stone after an impact with three different tools, as well as the development of the NSMS and determination of the specific work required to remove the stone embedded in the soil, and which outer plane was tangent to the soil surface.

## 2. Materials and Methods

### 2.1. Tools

**Figure 1:**
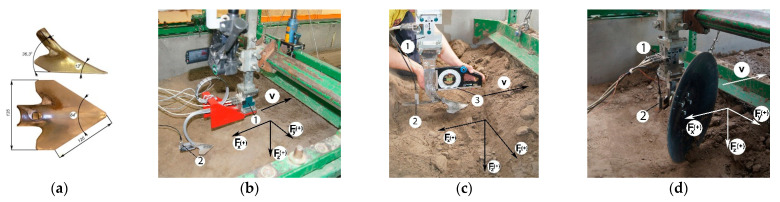
Tools used in the research: (**a**) duckfoot, (**b**) VCO-tine, (**c**) stiff tine, (**d**) a coulter with a smooth cutting edge; 1—strain gauge three-way force sensor CS3D, 2—three-way acceleration sensor MA 24.01 L N:628/10.3 × 160 m·s^–2^, 3—inclinometer and protector SLANT AL200, *F_x_*, *F_y_*, *F_z_*—component forces: draught, lateral and vertical, respectively, N, *v*—working speed of tool, m·s^–1^, (+)—positive direction.

In the study, a 135 mm-wide duckfoot attached to a Kongskilde VCO^TM^ flexible tine or a stiff tine and a coulter of a disc harrow (Figure 1) were used. Tools represent the working parts of the equipment for soil cultivation. The VCO tines are designed for shallow loosening of soil, tines with a stiff shaft are used in cultivators, and coulter discs are parts of disc harrows.

The constant of the VCO flexible tine determined in laboratory tests with a load of up to 500 N was 8.3 kN·m^–1^ (R^2^ = 0.99), and was greater than that of the flexible S-tine of 5.3 kN·m^–1^ [9]. The shaft of the stiff tine had a cross section of 10 × 60 mm. In both cases, the duckfoot’s clearance angle was 2°. The diameter of the coulter with a smooth cutting edge was 560 mm, and the rake angle was 23°.

The tools were attached to the frame of the tool carriage by means of a strain gauge three-way force sensor (CS3D, ZEPWN, Marki, Poland) to measure forces: draught *F_x_*, lateral *F_y_* and vertical *F_z_*. The adopted directions and turns of forces are shown in Figure 1.

### 2.2. Characteristic of Stones

The fieldstones and the pebbles were carefully selected in terms of shape (similar to a ball) and sorted into five dimensional groups with a weight ranging from 2.5 to 4.7 kg with five pieces in each group (row). The stones were painted white to increase the contrast with the soil and numbered. Each stone was weighed on electronic scales WLC 1/10.X2 (Radwag, Radom, Poland) with an accuracy of 0.1 g. After determining the planes and directions, using an angle bar, an aluminium strip, a digital level and a linear rule, the length, width and thickness of the stone were measured with an accuracy of 1 mm. The obtained results were used to calculate the equivalent diameter, sphericity and density of the stone [17]:

(1) $$d_{s}=\sqrt[{3}]{a_{s}b_{s}c_{s}},$$

(2) $$s_{f}=\left(\sqrt[{3}]{a_{s}b_{s}c_{s}}\right)/c_{s},$$

(3) $$\rho_{sc}=6\times 10^{6}m_{s}/\left(\pi a_{s}b_{s}c_{s}\right),$$

where *a_s_*, *b_s_*, *c_s_*, and *d_s_* are the thickness, width, length and equivalent diameter of the stone, respectively, in mm; *s_f_* is the sphericity of stone (dimensionless); *m_s_* is the mass of stone, in kg; and *ρ_sc_* is the computational stone density, in kg·m^–3^.

For control purposes, the stone’s density was determined by the pycnometric method according to the requirements of PN-EN 1936:2010-05P standard [18]:

(4) $$\rho_{s}=m_{s}\rho_{rh}/\left(m_{2}+m_{s}-m_{1}\right)\;$$

where *ρ_s_* is the real stone density, in kg·m^–3^; *m_s_* is the mass of a stone, in kg; *ρ_rh_* is the density of demineralized water, in kg·m^–3^, *ρ_rh_* = 1000 kg·m^–3^; *m*_1_ is the mass of a vessel with water and stone, in kg; and *m*_2_ is the mass of a vessel with water, in kg.

### 2.3. Soil Description

A fine loamy soil (PN-R-04033) with dry density of 1535 ± 11 kg·m^–3^ and moisture content of 9.4 ± 0.3% wb was used for tests (Table 1). The soil moisture content was determined by the drying-weighing method in accordance with the PN-ISO 11465:1999 standard. Soil moisture was controlled daily by taking a soil sample of 30 g and dried in a WPE 300 S RADWAG weigh-drier for 25 min. The accuracy of weighing the soil sample was 0.01 g. If, at the end of the day, the moisture decreased by 0.3%, water was added using the sprayers and the soil was loosened using the tines of a subsoiler. The soil compaction was 421 ± 13 kPa. Measurement of soil compaction to a depth of 0.15 m after soil compaction by a roller was carried out using the electronic penetrometer Penetrologger model 06.15.SA (Eijkelkamp Soil & Water, EM Giesbeck, The Netherlands) equipped with a cone of 30° apex angle and a base diameter of 20.60 mm.

### 2.4. Soil Bin Facility

**Figure 2:**
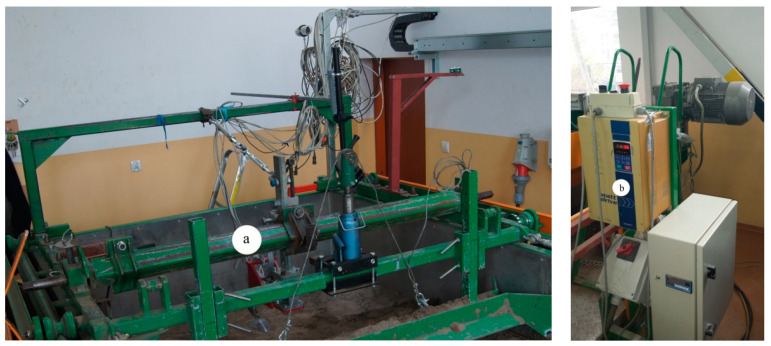
Tool carriage (**a**), drive and control system (**b**).

The experiments were performed in the soil bin of the Department of Biosystems Engineering, Institute of Mechanical Engineering, Warsaw University of Life Sciences. The soil was 0.6 m deep in the soil bin measuring 10 m × 2 m × 1 m [9]. The tool carriage running on rails was pulled in both directions by two steel ropes wound around a drum driven by a WAR 16 1M4 TF electric motor with a power of 22.0 kW and a rotational speed of 2920 rpm, by a reduction gear type FUA 85A 16 1M4 TF with a torque of 1084 N·m and a rotational speed of 194 rpm (Figure 2). The drive system was controlled by an inverter model V2500-0220 TFW1, with an 3PH 380-480 V 50/60 Hz 49A input and an 3PH 0–480 V 45 A output with a power of 22 kW, allowing us to change the frequency in the range of 0.1–400 Hz (Watt Drive, Markt Piesting, Austria).

The tool carriage was used to connect the tested tools, subsoiler tines, levelling scraper and compacting roller. 

The measurement system consisted of a laptop on which the test.comander^TM^ program (Gantner Instruments, Schruns, Austria) was installed for the configuration of the measurement system and data recording. It was connected to the test controller Q.station101 and measurement modules Q.A107, Q.A108, and Q.A116 (Gantner Instruments, Schruns, Austria) for the control, acquisition and manipulation of measured physical quantities. The sampling frequency during the research was 1000 Hz.

### 2.5. Soil Preparation

**Figure 3:**
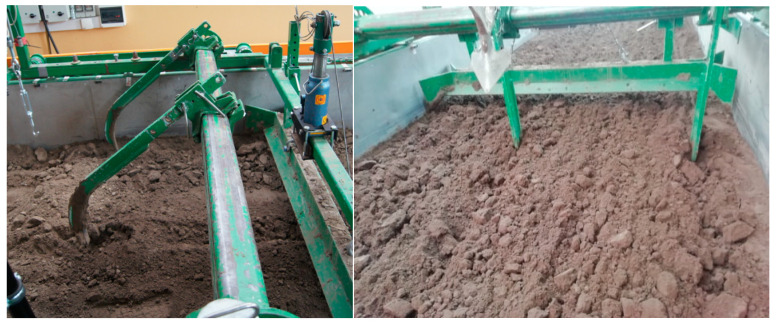
Subsoilers tines loosening the soil at a depth of 0.22 ± 0.02 m with a spacing of 155 ± 5 mm (for safety reasons, two tines were used in a single pass and their position was changed successively).

**Figure 4:**
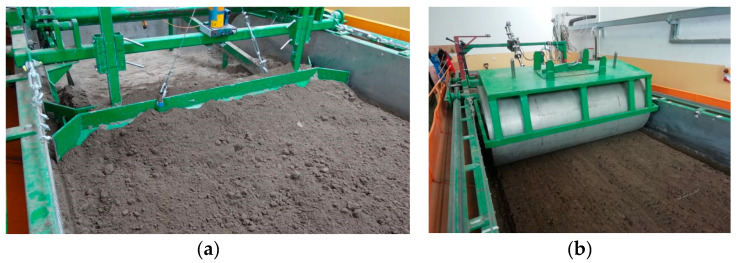
Levelling the soil with a two-sided scraper (**a**) and the first pass with the compacting roller (**b**) (without additional weights).

The soil was prepared to the assumed moisture level for a week before the tests. The soil was loosened to a depth of 0.22 ± 0.02 m with subsoil tines with spacing of 155 ± 5 mm (Figure 3). The outer surface of the soil was levelled with a double-sided scraper and compacted with a smooth roller weighing 360 kg in two directions (Figure 4). The compacting roller was transported to and from the soil bin using an electric crane ZSX-1000/3.0e with a lifting capacity of 1 t.

Levelling of the soil surface was checked by measuring vertical ordinates with a laser rangefinder (LDS 100-500P-S, Beta Sensorik) and soil compaction with an electronic penetrometer in five randomly selected places.

### 2.6. Location of Stones

The spacing of the rows planned along the soil bin and the columns forming a large-mesh grid was determined on the basis of preliminary tests to prevent stone and soil interaction between adjacent passes of tools. The stones were distributed by increasing mass (Table 2). For the flexible tine, stiff tine and coulter, 3, 4 and 5 rows were used, respectively, with row spacing of 0.50, 0.40 and 0.33 m, respectively. The distance of stones between the columns in the row was the same and was 0.80 m. Control tests with two stones in rows A and D were also performed (Table 2).

**Figure 5:**
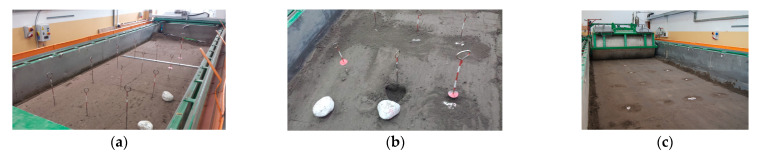
Procedure for placing stones: (**a**) planned grid for the placement of stones, (**b**) preparing a hole in the shape of a stone and placing the stones in the soil, (**c**) compaction of soil with stones.

The grid serving as the reference coordinate system formed nodes in which the soil was removed individually for each stone shape (Figure 5). The stones were embedded in the holes at a depth equivalent to the thickness of the stone so that the outer surface of the stone was tangent to the soil surface. After filling the small gaps with soil and manually compacting it around the stones, the soil with embedded stones was compacted with a roller with additional weights with a total weight of 520 kg, using a speed of 0.33 ± 0.01 m·s^–1^ and a single drive in both directions.

**Figure 6:**
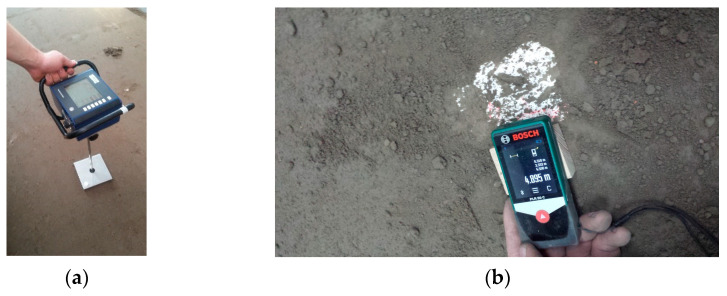
(**a**) Measurement of soil compaction by the electronic penetrometer Penetrologger model 06.15.SA and (**b**) stone placement in prepared soil and measurement of the initial position of the stone using the Bosch PLR 50C laser rangefinder.

Soil compaction was measured at five random points using an electronic Penetrologger (Figure 6). As the stones could move slightly after rolling, the actual coordinates of their position in relation to the side and end wall of the soil bin were determined using a Bosch PLR 50C laser rangefinder with an accuracy of 0.001 m (Figure 6). The measuring point was the intersection of the stone’s face in the direction of tool movement and a line passing halfway across the stone’s width.

### 2.7. Tool Attachment

The bracket fixing the tool to the carriage with a clamp allowed for setting the working depth. The tool angles and the working depth of the tools were set and monitored against the soil surface using a laser sensor, protractor and Bosch DNM 60 L electronic level. The clamp fixing the bracket with the frame of the carriage allowed for a rapid change in the position of the tools adapting to the spacing of the rows of stones. The duckfoot of the flexible and stiff tines was set along the axis of the stone row. The coulter had two positions. In one position, the cutting edge of the coulter coincided with the axis of the row of stones and cut the outer surface of the soil; this edge hit the stone halfway across its width, known as the coulter position in the axis of the stones. In a different position, the vertical plane crossing the center of the coulter coincided with the axis of the row of stones and the stones were cut with the soil, called the position of the disc encompassing the stone.

The working depths of the duckfoot with the flexible tine were 0.05 and 0.07 m, and those with the stiff tine were 0.07 and 0.10 m. The coulter worked at a depth of 0.07 and 0.10 m. The working speeds of the tines with the duckfoot were 0.83, 1.67 and 2.22 m·s^–1^, respectively, and 0.83 and 1.67 m·s^–1^, respectively, for the coulter’s edge aligner with the axis of the stones. The operating parameters were selected on the basis of actual requirements and initial tests, taking into account work safety and the protection of data measurement equipment. During the coulter operation already at the speed of 1.67 m·s^–1^, large overloads of the clamping system occurred when the cutting edge of the disc hit a stone. Therefore, the working speed of 2.22 m·s^–1^ was abandoned. The measurement system combination was summarized in Table 3.

### 2.8. The Displacement of Stones

**Figure 7:**
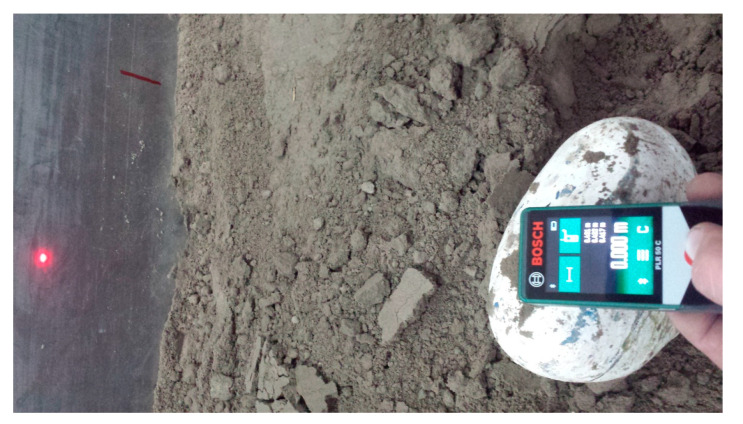
Measurement of the final position of the stone using the Bosch PLR 50C laser rangefinder.

After each working pass, the coordinates of the position of the shifted stones were measured with a laser rangefinder using the same reference planes as for the initial position of the stones: the side and end wall of the soil bin (Figure 7). The stones were successively removed from the soil bin and the scattered soil was removed (after each pass) so that the lumps and loose soil would not interfere with the test of the next row of stones.

Based on the measured coordinates of the stone position before and after the contact with the tool, the distance travelled by the stone *s_s_*, the slope factor of the straight line for stone movement *k_s_*, and the angle (value modulus) of stone movement relative to the direction of the tool movement *α_s_* were calculated.

(5) $$s_{s}=\sqrt{{\left(x_{e}-x_{s}\right)}^{2}+{\left(y_{e}-y_{s}\right)}^{2}}$$

(6) $$k_{s}=\tan\left(\alpha_{s}\right)=\left(y_{e}-y_{s}\right)/\left(x_{e}-x_{s}\right)\;$$

(7) $$\alpha_{s}=\left|\tan^{-1}\left[\left(y_{e}-y_{s}\right)/\left(x_{e}-x_{s}\right)\right]\right|$$

where *s_s_* is the distance travelled by the stone after impact, in m; (*x_s_*,*y_s_*) are the coordinates of the initial position of the stone, in the direction of movement of the tool and laterally, respectively, in m; (*x_e_*,*y_e_*); *k_s_* is the slope factor of the straight line for stone movement; and *α_s_* is the modulus of the angle for stone movement relative to the direction of the tool movement, in °.

The movement of tools and stones was filmed with three digital cameras. The Digital Still Camera Sony Cyber-Shot DSC-HX100V 16.2 MP Exmor R CMOS with Carl Zeiss Vario-Tessar 30x Optical Zoom Lens and Full HD 1080 Video (Sony Poland Ltd., Warsaw, Poland) moved with the tool carriage and filmed the work of the tool from the side. The Sony HDR-XR500V 12.0 MP digital camera (Sony Poland Ltd., Warsaw, Poland) was permanently installed on the rear wall of the soil bin and filmed the work of the tool at the back. The Xiaomi YI Action camera (YI Poland Ltd., Warsaw, Poland) was suspended from the ceiling and filmed the work of the tool from above. Time-lapse analysis of the photos allowed for the interpretation of the movement of the tools and each individual stone during and after the impact.

The position of the stone after the interaction with the tool was described using the developed Numerical Stone Movement Scale (*NSMS*) (Table 4).

### 2.9. Specific Work Required to Remove the Stone Embedded in the Compacted Soil

**Figure 8:**
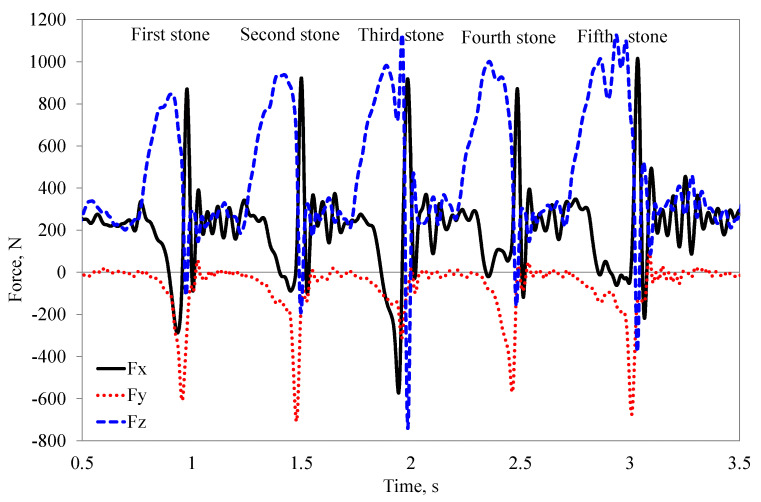
An example of recorded forces *F_x_*, *F_y_* and *F_z_* in three mutually perpendicular directions during the operation of the flexible tine with the duckfoot tool at the depth of 0.07 m and a speed of 1.67 m·s^–1^ (repeated peaks indicate force dynamics before and after contact with the stone).

To calculate the force required to remove the stone embedded in compacted soil, values around the maximum force consisting of 20 measurement points were selected (Figure 8). 

Based on the three components of the forces the resultant force was determined. Additionally, the mass of the stones was different; therefore, the specific force related to the mass of the stone was calculated for the purpose of comparative analysis.

(8) $$F_{a}=\sqrt{F_{x}^{2}+F_{y}^{2}+F_{z}^{2}}/m_{s}$$

The directional coefficient of the specific force *k_Fxy_*, resultant in the horizontal plane between the *F_x_* and *F_y_* forces, and the angle (modulus) of the resultant force in the horizontal plane relative to the direction of the tool movement *α_Fxy_* were calculated.

(9) $$k_{Fxy}=F_{y}/F_{x}$$

(10) $$\alpha_{Fxy}=\left|\tan^{-1}\left(F_{y}/F_{x}\right)\right|$$

The specific work required to remove the stone embedded in compacted soil *L_s_* was calculated using the formula:

(11) $$L_{s}=F_{a}s_{s}$$

where *F_m_* is the resultant specific force, in N·kg^–1^; *F_x_*, *F_y_* and *F_z_* are component forces, namely draught, lateral and vertical, respectively, in N; *m_s_* is the mass of the stone, in kg; *k_Fxy_* is the directional coefficient of the specific force, resultant in the horizontal plane between the *F_x_* and *F_y_* forces; *α_Fxy_* is the angle of the resultant force in the horizontal plane relative to the direction of the tool movement, in °; *L_s_* is the specific work required to remove the stone embedded in compacted soil, in J·kg^–1^; and *s_s_* is a distance travelled by the stone, in m.

### 2.10. Statistical Analysis

The characteristics of the stone expressed as mass *m_s_*, equivalent diameter *d_s_*, sphericity *s_f_*, computational *ρ_sc_* and real *ρ_s_* densities were determined by the mean and standard deviation.

On the basis of the statistical analysis, the influence of the tool type, depth *a* and working speed *v* on the distance travelled by the stone after the impact with the tool *s_s_*; the slope factor of the straight line for stone movement *k_s_*; the modulus of the angle for stone movement relative to the direction of the tool movement *α_s_*; the Numerical Stone Movement Scale *NSMS*; the directional coefficient of the specific force resultant in the horizontal plane *k_Fxy_*; the angle of the resultant force in the horizontal plane relative to the direction of the tool movement *α_Fxy_*; and the specific work required to remove the stone embedded in compacted soil *L_s_* was determined.

The statistical analysis of the results was performed using the Statistica^TM^ v.13.3 software (StatSoft Poland Ltd., Cracow, Poland). A detailed analysis of mean values was performed using the Tukey test. The significance of differences between mean values was checked at the significance level *p* = 0.05.

## 3. Results and discussion

### 3.1. Characteristics of Stones

The mean mass of the stones in the subsequent columns increased by about 30% and the mean values formed individual homogeneous groups of masses from 2.52 to 4.72 kg (Table 5). The sets of five stones in the individual columns were carefully selected in terms of the mass distribution as the relative error of the weight distribution was in the range of 3.4–5.6%.

The equivalent diameter of the stone increased proportionally to the mass from 0.123 to 0.154 m. The proof is the very high value of the correlation coefficient between these physical quantities, r = 0.927 (almost full correlation). Under natural conditions, the diameters of the stones vary greatly from 0.002 to 0.200 m, while stones with a diameter of 0.063–0.200 m are classified as coarse [19].

Stones with a smaller mass and diameter were characterized by a greater sphericity than larger stones, as evidenced by the negative values of correlation coefficients: –0.462 and –0.451, respectively (average correlations). There were some inconsistencies in columns B and C, because sphericity increased from 0.825 to 0.856. The smallest stones were very close to a sphere, *s_f_* = 0.900. The sphericity of the stones in columns A–C was similar and was greater than that of the stones in columns D and E.

The computational densities of the stones were in the range of 2458–2861 kg·m^–3^, and the overall mean was 2633 kg·m^–3^ with a relative spread error of 3%. An even more close range was obtained for the real stone density of 2764–2913 kg·m^–3^, with an overall mean of 2831 kg·m^–3^ and a relative spread error of 1%. This result is evidence of the careful selection of the stones in terms of the dimensions and types of the stones’ parent rocks. The average specific density of limestones is in the range of 1790–2920 kg·m^–3^. According to the American Society for Testing and Materials (ASTM D 153), the density of granite stones is 2560–2700 kg·m^–3^ [20]. Compared to the bulk density of soil of 1300 kg·m^–3^, the bulk density of the stones is 2650 kg·m^–3^ [19]. There are no physical properties of stones in the available literature. In the meta-data analysis, it was noted that among the most important parameters characterizing the soil, in many cases stone content and soil moisture were missed [19]. These authors concluded that a proper soil texture analysis cannot be performed if the stone content is missing.

### 3.2. Movement of Stones Embedded in the Soil under the Influence of the Forces of Working Elements

Based on the analysis of the correlation of the assumed parameters [21], it was found that the angle of the resultant force in the horizontal plane relative to the direction of the tool movement *α_Fxy_* almost fully correlates (r = 0.979) with the directional coefficient of the resultant force in the horizontal plane *k_Fxy_* (Table 6). 

Therefore, the angle *α_Fxy_* was removed from further analysis. The other six parameters are sufficient to fully characterize the movement of stones embedded in the soil. The values of these parameters in relation to the tested factors are summarized in Table 7.

**Figure 9:**
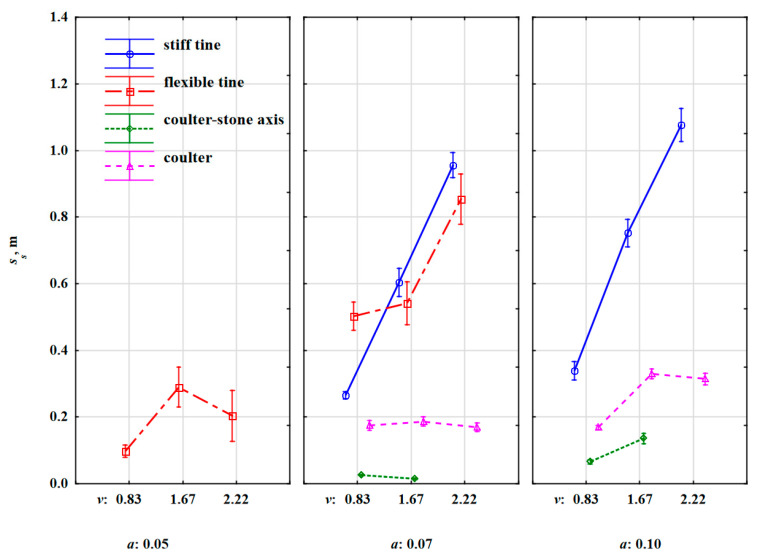
Stone displacement distance due to forces of the stiff tine, flexible tine, coulter-axis stones and coulter working at different working depths and speeds.

The greatest distances travelled by the stones were caused by the extortions from the stiff tine (Table 7). This distance strongly depended on the working speed and to a smaller extent on the working depth (Figure 9). 

At the speeds of 0.83, 1.67 and 2.22 m·s^–1^ and the depth of 0.07 m; *s_s_* was 0.26, 0.60 and 0.96 m, respectively, and at a depth of 0.10 m, it was greater by 12–31% and was 0.34, 0.75 and 1.08 m, respectively. At a higher working speed of the tool, the stone obtained greater kinetic energy and was thrown out at a greater distance than at a lower speed. This effect of working speed is more visible for sandy soils [9].

**Figure 10:**
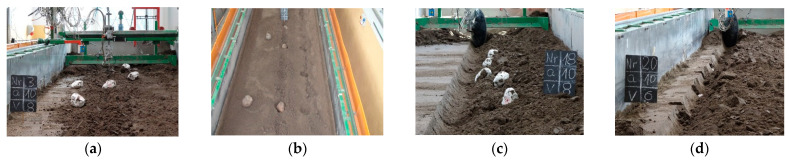
Displaced stones for exemplary working parameters during the operation of the: (**a**) stiff tine (*a* = 0.10 m, *v* = 2.22 m·s^–1^), (**b**) flexible tine (*a* = 0.05 m, *v* = 2.22 m·s^–1^), (**c**) coulter (*a* = 0.10 m, *v* = 2.22 m·s^–1^), (**d**) coulter-axis stones (*a* = 0.10 m, *v* = 1.67 m·s^–1^).

After overcoming the inertia forces, the stone embedded in the soil achieved acceleration, which was the greater the higher the working speed was, and this resulted in a greater distance over which the stone was thrown out by the stiff tine (Figure 10a). The extent of cracking in the soil layer at a higher working depth of the tool was greater, and the movement of the stone began earlier, even before contact with the tool, obtaining higher kinetic energy than at a shallower depth.

These results correspond to greater soil dispersion at higher working depths [10]. However, in the tests performed on sand in relation to a 0.038 m wide stiff tine with a soft steel hemisphere at a small speed range of 0.10–1.0 m·s^–1^, no effect of speed on the movement of the hemisphere before contact with the tine was found [16]. For both working depths of 0.07 and 0.10 m, the beak of the duckfoot sweep was below the middle of the stone’s thickness of 0.056 m. This condition was met even for the thickest stone of 0.12 m, for which the centre of thickness was 0.06 m. In these conditions, the movement of the stone began with the wedge of the cut-off soil layer even before the contact between duckfoot and the stone. Such an extent of soil fracture is characteristic of wide tools. Wide tools fracture the soil upward and forward and tend to loosen the soil in a crescent shape [22]. Wide tools are those whose width is greater than the working depth, i.e., the blade shape factor (depth/width) is less than 1.0 [23]. A duckfoot with a width of 135 mm working at a depth of 0.05–0.10 m can be classified as a wide tool, because the blade shape factor is 0.37–0.74. For wide tools, only forward soil movement is considered, and this problem is analysed in 2D [24].

The fracture range of the soil wedge stopped for a moment in front of the stone during the work of the stiff tine at a greater depth. As the tool continued to move, the stone began to move with the soil wedge embracing the stone, and at the same time the stone pushed the soil wedge created by the stone forward, which deepened the cracking of the soil compared to the set working depth for the duckfoot. The soil in front of the stone was not cut but pushed, as by a very blunt edge of a blade [25]. With further pressure of the tool, the stone embedded in the soil achieved high acceleration and was thrown out to a distance proportional to the working speed of the tool.

During the work of the flexible tine at a working depth of 0.05 m, the beak of the duckfoot was at a depth close to the middle of the stone’s thickness. Under these conditions, the beak of the duckfoot hit the stone above the centre of the stone’s thickness or only slightly below, and the movement of the stone began without giving an initial speed through the cut soil. Under these conditions, the stone was pushed parallel to the soil for a while. As a result of the resistance, the flexible tine bent backwards, suppressing the impact. In many cases, the duckfoot attached to the flexible tine moved over the outer surface of the stone, causing it to move, rotate, or be slightly displaced (Figure 10b). The low kinetic energy obtained by the stone contributed to the small distance travelled by the stone (0.10–0.29 m). As the centre of the thickness of the stones was in the range of 0.04–0.07 m, the place of impact of the duckfoot’s beak with the stone was variable and hence the changes in *s_s_* vs. the working speed of the tool were inconclusive (Figure 9). Increasing the working depth of the flexible tine to 0.07 m resulted in a change in the duckfoot–stone relationship, and *s_s_* increased with the working speed of the tool. The distance travelled by the stone after the reaction with the flexible tine was 10–11% smaller than with the stiff tine in the speed range 1.67–2.22 m·s^–1^. The flexible tine, encountering the resistance of the stone, was deflected back, absorbing some of the energy from the impact with the stone; hence, the kinetic energy transmitted by the flexible tine was smaller than that of the stiff tine. At the lowest working speed of the tool, 0.83 m·s^–1^, the vibrations of the flexible tine were the lowest and the tine’s reactivity to the resistance of stone embedded in the soil was greater than at higher working speeds. The flexible tine tilted back, accumulating greater kinetic energy, and in the acceleration in the forward direction transferred energy to the stone which under its influence was moving. At the lowest working speed, the oscillations of the flexible tine were smaller [9] and the impact of the flexible tine with the stone was more precise than at higher operating speeds. During the work of the flexible tine at the speed of 0.83 m·s^–1^, the distance travelled by the stone was 0.50 m and was 92% greater than for the stiff tine. These explanations are based on the description of the cyclic reaction of a flexible tine working in non-stony soil [15]. The peak load refers to the rearward hitting, and then the soil was loaded on the deviated tine and, after exceeding the strength, the soil crack and the tool entered the cracked soil, reducing the draught force. In the case of a stone embedded in the soil, the effect of cracking the soil was smaller as the stone provided additional resistance and possibly a greater impulse of force caused increase in the distance travelled by the stone.

During the work of the coulter covering stones, the stone displacement distances were small, and at the working depth of 0.07 m, they did not exceed 0.20 m, regardless of the working speed (Figure 9). At a working depth of 0.10 m, the displacement of the stones increased almost twice, but only at higher working speeds of 1.67–2.22 m·s^–1^ when the stones obtained higher kinetic energy. The stones embedded in the soil were displaced with the cut off furrow and at a lower working depth (0.07 m), the stones were partially recessed into the furrow at a greater working depth (0.10 m), and they were moved to outside the cut furrow (Figure 10c).

Compared to the coulter covering stones, the work of the coulter coinciding with the axis of the row of stones was more fine, because the cutting edge of the coulter only slightly moved the stones (Figure 9) in the direction of movement and pressed the stones into the soil (Figure 10d). Most often, the coulter rolled centrally on the stones or with an offset relative to the centre of the width of the stones, pressing the stones into the face or much less frequently moving the stones towards the cut furrow. The cutting edge of the coulter met the embedded stone in the compacted soil. This observation was very critical, as there were temporary but sudden overloads of the coulter disc and the clamping element which were transferred to the tool. In real conditions, these overloads can cause structural parts of the tool to break or bend. Therefore, agricultural implements with coulters used for the cultivation of stony soils should be (and most often are) equipped with elements protecting against their damage.

### 3.3. Stone Movement Direction k_s_, Angle of Stone Movement α_s_ and Numerical Stone Movement Scale NSMS

**Figure 11:**
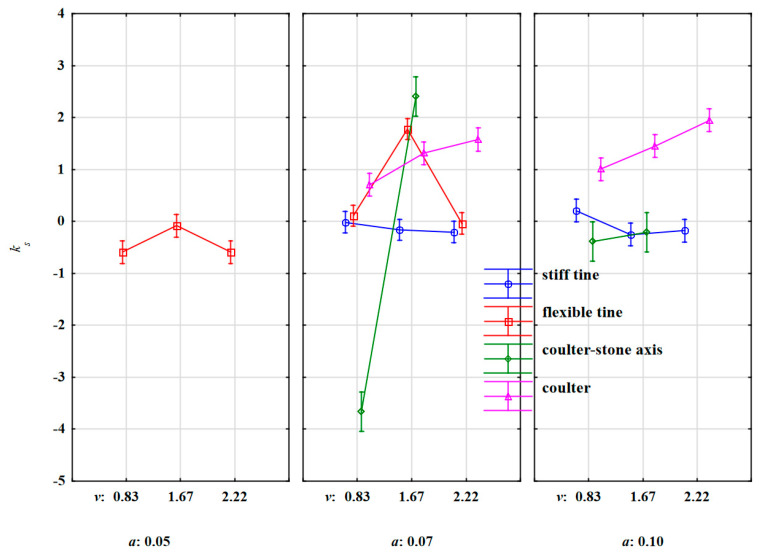
Slope factor of the straight line for stone movement *k_s_* discarded under the extortions of the stiff tine, flexible tine, coulter-axis stones and coulter working at varied depths and working speeds.

The values of the directional coefficients of stone movement under the action of stiff and flexible tines were strongly differentiated (Figure 11). The large standard deviation values (Table 7) were also deemed ambiguous because *k_s_* was both positive (stone movement to the right in relation to the tool movement direction) and negative. Therefore, it can be concluded the direction of motion was random and resulted from the random distribution of reactions in the place of impact of the duckfoot with the stone.

During the work of the coulter impacting the stone, the slope factor of stone movement increased with the working speed (Figure 11). At a higher working depth and a higher working speed of the tool, the stone was pushed forward in the direction of the tool’s movement, and then moved along with the soil to the right. Together with the working speed of the tool, the stone obtained higher kinetic energy and was moved with an increasing slope factor of stone movement. A positive value of the slope factor for the stone movement (1.33) during the work of the coulter impacting the stone indicates the unambiguous direction of stone displacement, as evidenced by the relatively small value of the standard deviation 0.02.

Similarly, during the work of the coulter-axis stones, the values of the slope factor for stone movement had a strongly different trend for both working depths. The values of these factors increased with the working speed of the tool; faster for a smaller working depth of 0.07 m than for 0.10 m (Figure 11). The highest displacement of stones to the left in relation to the direction of movement was recorded during coulter working at the depth of 0.07 m and the working speed of 0.83 m·s^–1^. As mentioned, the displacement of the stones to the left was due to the fact that the coulter pressed the stone into the soil and simultaneously moved the stone towards the face (Figure 10d). The highest slope factors for stone movement to the right in relation to the direction of the coulter-axis stones were recorded at the working depth of 0.07 m and the working speed of 1.67 m·s^–1^. The work of the coulter-axis stones was more stable at the depth of 0.10 m. In summary, the values of slope factors confirm the observed movement of stones under the influence of the forces of working elements (Figure 10).

The slope factor for stone movement is an important parameter indicating the behaviour of stones hit by the working element. In practice, it is also important to use the absolute value of the angle of stone movement with respect to the direction of the tool’s movement.

**Figure 12:**
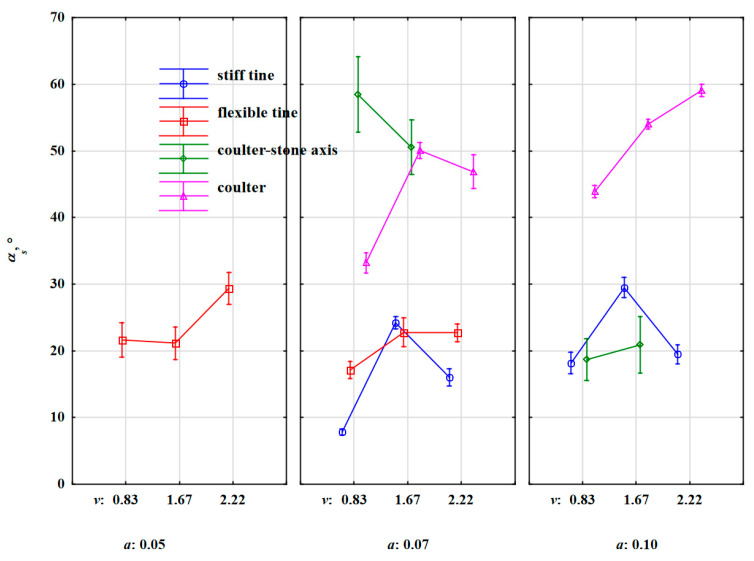
The angle for stone movement relative to the direction of the tool movement *α_s_* under the extortions of the stiff tine, flexible tine, coulter-axis stones and coulter working at varied depths and working speeds.

During the work of the flexible tine at a depth of 0.05 m, the stones were displaced at angles of 21°–29° (Figure 12). Increasing the working depth to 0.07 m caused only a slight reduction in these angles to 17°–23°.

The stiff tine working at a depth of 0.07 and 0.10 m threw out stones at similar angles within the range of 8°–29°.

The impact of the working speed on the angle of stone movement for both tines was ambiguous, which confirms the random nature of the stone’s reaction to the extortions from both the flexible and the stiff tines. However, the standard deviation was 43% greater for the flexible tine, which indicates a larger dispersion of values (Table 7).

High values of stone movement angles were recorded during the work of the coulter covering stones especially at a greater working depth (0.10 m), *α_s_* = 44°–59°. The stone movement angles recorded during the work of the coulter-axis stones at a depth of 0.07 m, *α_s_* = 51°–58°, were also in this range. Increasing the depth to 0.10 m caused a significant reduction in the angles of stone movement, *α_s_* = 19°–21°.

Compared to the slope factor for the stone movement, the angle of stone movement was correlated to a greater degree with the factors and between parameters (Table 6). The correlation was average, but negative between the angle of movement of the stones and the distance travelled by the stones, r = –0.311. The correlations were high between the angle of stone movement and the directional coefficient of the specific force resultant in the horizontal plane *k_Fxv_* and the angle of the resultant force in the horizontal plane relative to the direction of the tool movement, because the correlation coefficients were 0.485 and 0.506, respectively. The higher the distance travelled by the stone, the smaller the angle of stone movement, i.e., the stones were directed more towards the tool’s movement. The positive correlation coefficients of the angle for stone movement *α_s_* with *k_Fxy_* and *α_Fxy_* indicate one-way reactions of these parameters during the impact of the working element with the stone. The direction of extortions from the tool influenced the angle of the stone movement in relation to the tool movement direction.

**Figure 13:**
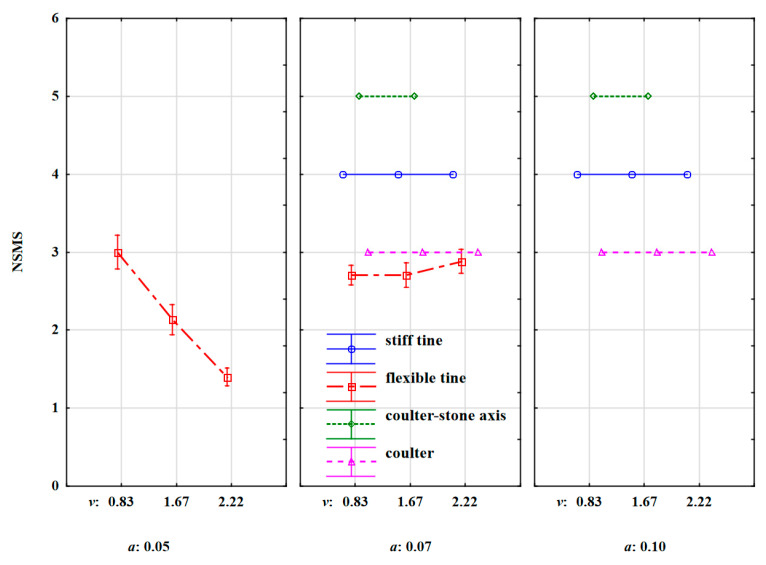
Numerical Stone Movement Scale (*NSMS)* determining the behaviour of the location of stones shifted under the influence of forces coming from the stiff tine, flexible tine, coulter-axis stones and coulter working at varied depths and working speeds.

Although the behaviour of the stones under the influence of the tools was characteristic for a given type of tool, the most diversified stone movement was recorded during the work of the flexible tine working at a depth of 0.05 m. Interestingly, at the lowest working speed of 0.83 m·s^–1^ the stones were moved (Figure 13). At the speeds of 1.67 and 2.22 m·s^–1^, the stones were properly rotated and moved as strong oscillations and backward deflections of the tine contributed to the penetration and movement of the duckfoot along the outer surface of the stones. For these three depths, the *NSMS* values were 3.0, 2.1, and 1.4, respectively. During the work of the flexible tine at a greater working depth of 0.07 m, the stones were moved regardless of the working speed of the tool, and the *NSMS* value was 2.7–2.9. During the work of the stiff tine and the coulter covering the stones and the coulter-axis stones, the stones were thrown out, moved and pressed into the soil, respectively, regardless of the speed and working depth of the tool. The *NSMS* values were 4.0, 3.0, and 5.0, respectively (Figure 13). The developed *NSMS* confirms the previously described behaviour of stones under the influence of tool extortions, and this is the basis for assessing the movement of stones hit by the tested tools. It can be useful for assessing stone movement under the impact of other types of tools or working elements.

### 3.4. The Directional Coefficient of the Specific Force Resultant in the Horizontal Plane k_Fxy_ and Specific Work Required to Remove the Stone Embedded in Compacted Soil L_s_

**Figure 14:**
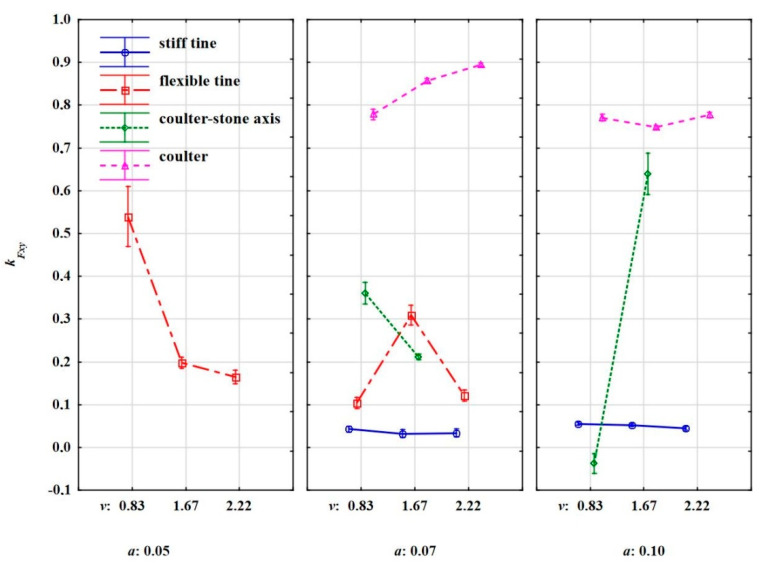
The directional coefficient of the specific force resultant in the horizontal plane *k_Fxy_* as a reaction when the elements collide with stone: stiff tine, flexible tine, coulter-axis stones and coulter working at varied depths and working speeds.

The directional coefficient of the specific force resultant in the horizontal plane *k_Fxy_* allows for the interpretation of the direction of the push on the stone when the tool collides with the stone. In almost all cases, the resultant force in the horizontal plane was directed to the right, as evidenced by the positive values of the directional coefficient (Table 7, Figure 14).

During the work of the flexible tine, the direction of the resultant force was the most ambiguous (Figure 14). At the working depth of 0.05 m, a certain trend decreasing with the working speed could be observed. At higher working speeds of 1.67–2.22 m·s^–1^ the impacts between the tool and stone were more central. The most symmetrical impacts were recorded during the work of the stiff tine, because for both working depths of 0.07 and 0.10 m, and regardless of the working speed the values of the directional coefficient were small and in a narrow range of 0.03–0.06. They were equivalent to very small angles of deviation of the resultant force from the direction of the tool movement which were 1.8°–3.0°.

The highest values of the directional coefficient of the resultant force were recorded during work of the coulter covering the stone. During the work of the coulter covering the stone at the depth of 0.07 m, *k_Fxy_* = 0.78–0.89, the angle of deviation of the resultant force was 37.9°–41.8°. At this working depth, the directional coefficient of this force slightly increased with the working speed. At a higher working depth of 0.10 m, *k_Fxy_* was slightly lower than at *a* = 0.07 m, and practically did not depend on the working speed and was 0.75–0.78. High values of the directional coefficient of the resultant force during the work of the coulter covering the stone were related to the rake angle of the coulter of 23° in relation to the direction of movement and the unambiguous character of the movement of stones embedded with the soil.

A completely different response of the direction of the resultant force in the horizontal plane was during the work of the coulter-axis stones when the edge of the disc assumed a random position in relation to the stone but pressed down on the stone. The consequences of these random contacts of the coulter edge with stones were ambiguous changes and large differences in the *k_Fxy_* values, especially at a greater working depth of 0.10 m as the values varied from –0.04 to 0.64 (Figure 14).

The *k_Fxy_* values were average, but inversely correlated with *s_s_* and the specific work required to remove the stone embedded in the soil *L_s_*, and the correlation coefficients were –0.365 and –0.332, respectively (Table 6). The more the resultant force in the horizontal plane was deviated from the direction of movement, the shorter the distance the stone travelled and the smaller the specific work that was required to remove the stone.

**Figure 15:**
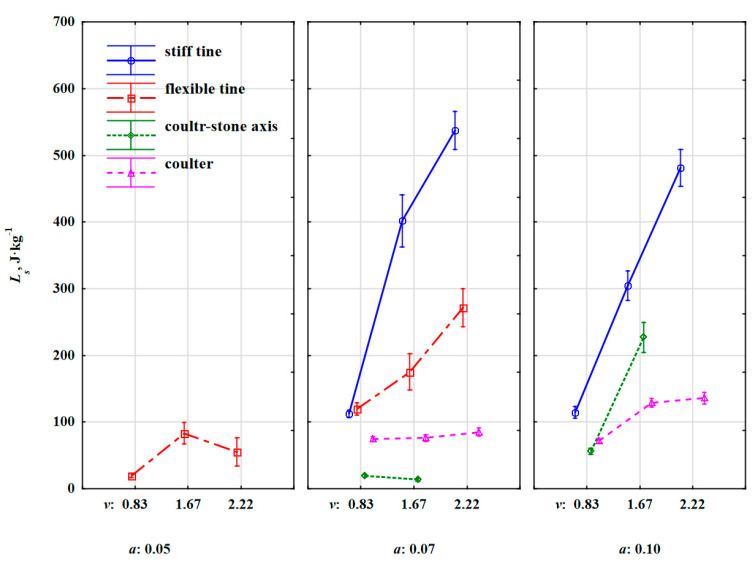
Specific work required to remove the stone embedded in compacted soil *L_s_* depending on the stiff tine, flexible tine, coulter-axis stones and coulter working at varied depths and working speeds.

Changes in the specific work required to remove the stone embedded in the soil *L_s_* vs. speed and working depth (Figure 15) were similar to changes in *s_s_* (Figure 9). The proof is the high value of the correlation coefficient between these parameters, r = 0.863 (very high correlation) (Table 6).

One of the significant differences between *L_s_* and *s_s_* were the values of this work for the flexible tine working at a depth of 0.07 m in relation to the values obtained for the stiff tine. At the lowest working speed of 0.83 m·s^–1^, specific work had similar values for both flexible and stiff tines: 119 and 114 J·kg^–1^, respectively. Compared to the relation of the distance travelled by stones for both tines (Figure 9), a significant reduction in this work for the flexible tine was due to the lower value of the resultant force needed to remove the stone, which is a positive feature for flexible tines [15]. This trend of significantly lower specific work values for the flexible tine compared to the stiff tine extended to higher working speeds of 1.67 and 2.22 m·s^–1^, because the decreases in the values of this specific work were 74% and 77%, respectively.

The specific work *L_s_* for the stiff tine in relation to the working speed maintained a similar trend as the characteristic of the distance travelled by the stone, while higher values of *L_s_* were recorded for a smaller working depth of 0.07 m than for a greater depth of 0.10 m. The value of this specific work was more influenced by the specific resultant force (in N·kg^–1^) pressing on the stone embedded in the soil than by the distance *s_s_*. At a smaller depth of 0.07 m, the impact of the duckfoot beak with the stone was close to half of its thickness and the soil in front of the stone’s face was compressed, which suppressed the stone’s movement, but also generated a greater resistance force and consequently more specific work was needed to remove the stone than at a greater depth of 0.10 m. At the working depth of 0.10 m, the beak of the duckfoot was below the lower half of the stone thickness. The propagation of soil fracture starting from the duckfoot tip set the stone in motion without a sudden impact of the duckfoot with the stone. In addition, the stone was pushed upwards with the soil wedge at a shear angle without the additional compressing of the soil by a stone in a horizontal direction. Therefore, at the working depth of 0.10 m, the specific work required to remove the stone was on average 14% lower than at the depth of 0.07 m. The highest values of specific work were achieved at the working speed of 2.22 m·s^–1^, which were 537 and 481 J·kg^–1^, respectively.

The characteristics of specific work needed to remove the stone embedded in the soil by the coulter were similar to those of the distance travelled by the stone. Higher differences were found when the coulter was working in the axis of the stone row at a greater working depth of 0.10 m, which was caused by a very dynamic reaction when the disc edge hit the stone and forced it into the soil. Increasing the working speed of the coulter from 1.67 to 2.22 m·s^–1^ contributed to a four-fold increase in the specific work *L_s_* from 56 to 227 J·kg^–1^. This result is evidence of the enormous overload that occurs when rolling over the stone while pressing it into the compacted soil.

The specific work required to remove the stone embedded in compacted soil related to the mass of stone is a good parameter for comparing the effects of tool work in stony soil. Since the specific work contains the specific force as the reaction of the tool to the impact with the stone and the distance travelled by the stone after the impact, *L_s_* is a valuable parameter describing the relationship between the tool and the stone embedded in compacted soil. Further research may be conducted on the relationship between the tool and the stone embedded in loose soil or on the surface of the soil to obtain an insight into the differences in the phenomena occurring during and after the tool collides with the stone. It would also be advisable to study the stone’s movement trajectory for different conditions of stone location.

## 4. Conclusions

Spherical-like silicate stones with a sphericity of 0.79–0.90 and a mass of 2.52–4.72 kg were embedded in the compacted fine loamy soil (con index = 421 kPa) at a depth equivalent to its thickness. The stones formed a grid of three to five rows and five columns with dimensions of 0.33–0.50 m × 0.8 m, respectively. The stones were hit by stiff and flexible tines with a 135 mm wide duckfoot and a 560 mm diameter coulter at rake angle of 23°. The tines were aligned with the stone row. The coulter covered the stone or the cutting edge of the coulter disc hit at the half of the width of the stone. The tools worked at various parameters, in the depth range of 0.05–0.10 m and a speed of 0.83–2.22 m·s^–1^. The behaviour of the stones after the impact was filmed with three cameras from the side, back, and top. The characteristics of stone movement were specific to the type of tool and were described by the Numerical Stone Movement Scale. After the impact with the stiff tine, the stones were thrown over distances of 0.26–1.08 m proportional to working speed. The flexible tine working at a shallower depth of 0.05–0.07 m endured high vibrations and hit the stones randomly; most often above or near the centre of the stone thickness of 0.056 m. Depending on the accidental contact, the stones were slightly moved and turned, and during a central impact they were moved over a distance of 0.50–0.85 m. Stones covered by the coulter were displaced with the soil over a distance of 0.17–0.33 m. The coulter hitting the cutting edge pressed the stones into the soil or the face, moving them slightly at a distance of 0.01–0.14 m. The direction and angles of stone movement under the influence of the tool forces were ambiguous. The specific work required to remove the stone was a reflection of the distance travelled by the stone as well as the specific force which greatly contributed to the increase in the difference of this work between the stiff and flexible tines.

The described relationships between the tool and stone embedded in the compacted soil should be extended to stones embedded in loose soil or located on the soil surface. A study of the stone’s trajectory and the forces induced in the tool would broaden the knowledge of the tool–stone relationship during or after the impact.

## Figures and Tables

**Table 1:** Physical properties of the experimental soil.

Parameters	Values
Soil type	Fine loamy soil
Clay content (<0.002 mm) (%)	2
Silt content (0.002–0.05 mm) (%)	36
Sand content (>0.05 mm) (%)	62
Dry density for tests (ρ, kg m^–3^)	1535 (11)
Cone index (CI, kPa)	421 (13)
Moisture content (wb) for tests (mc, %)	9.4 (0.3)
Angle of internal friction (φ, ^o^)	37
Angle of soil-metal friction (δ, ^o^)	24
Cohesion (c, kPa)	17
Adhesion (c_a_, kPa)	10

Standard error in parenthesis.

**Table 2:** Mass of stones used in tests.

Rows	Mass of Stones, kg				
	Columns				
	A	B	C	D	E
1	2.46	2.98	3.70	4.26	4.64
2	2.68	3.06	3.72	4.28	4.70
3	2.58	2.96	3.60	4.30	4.84
4	2.56	2.96	3.86	4.22	4.52
5	2.32	2.92	3.70	4.42	4.92

**Table 3:** Design of experiments.

Tool	Flexible Tine															Stiff Tine															Coulter													
Measuring system	soil						A and D stone			5 stones						soil						A and D stone			5 stones						soil				5 stones						5 stones axis			
Depth *a* (m)	0.05			0.07			0.07			0.05			0.07			0.05			0.10			0.07			0.07			0.10			0.07		0.10		0.07			0.10			0.07		0.10	
Speed *v* (m·s^–1^)	0.83	1.67	2.22	0.83	1.67	2.22	0.83	1.67	2.22	0.83	1.67	2.22	0.83	1.67	2.22	0.83	1.67	2.22	0.83	1.67	2.22	0.83	1.67	2.22	0.83	1.67	2.22	0.83	1.67	2.22	0.83	2.22	0.83	2.22	0.83	1.67	2.22	0.83	1.67	2.22	0.83	1.67	0.83	1.67
Number of stones	0						2			5						0						2			5						0				5									
Repetitions	1						1			3						1						1			3						1				3									

**Table 4:** Numerical Stone Movement Scale—*NSMS.*

Stone Reaction after Contact with the Working Element	*NSMS*
Intact	0
Moved	1
Turned	2
Displaced	3
Flanged	4
Pressed (into the soil)	5

**Table 5:** Characteristics of stones; mean values and standard deviations.

Columns	Mass *m_s_*, kg	Equivalent Diameter *d_s_*, m	Sphericity *s_f_*	Computational Density *ρ_sc_*, kg·m^–3^	Real Density *ρ_s_*, kg·m^–3^
*p*-value	<0.0001	<0.0001	<0.0001	<0.0001	<0.0001
A	2.52 ^a*^ ± 0.14	0.123 ^a^ ± 0.001	0.900 ^d^ ± 0.004	2612 ^b^ ± 22	2913 ^c^ ± 6
B	2.98 ^b^ ± 0.05	0.129 ^b^ ± 0.001	0.825 ^c^ ± 0.012	2669 ^b^ ± 26	2833 ^b^ ± 12
C	3.72 ^c^ ± 0.09	0.140 ^c^ ± 0.001	0.856 ^b^ ± 0.011	2567 ^ab^ ± 31	2810 ^ab^ ± 9
D	4.30 ^d^ ± 0.07	0.142 ^c^ ± 0.001	0.794 ^ab^ ± 0.005	2861 ^c^ ± 37	2837 ^b^ ± 22
E	4.72 ^e^ ± 0.16	0.154 ^d^ ± 0.001	0.785 ^a^ ± 0.005	2458 ^a^ ± 18	2764 ^a^ ± 10

* Different letters in each column represent a significant difference at *p* < 0.05 using Tukey’s test.

**Table 6:** The matrix of correlation coefficients for factors and parameters characterizing the movement of stones under the influence of forces of working elements.

Parameter	Tool ^*^	*a*	*v*	*m_s_*	*s_s_*	*L_s_*	*k_s_*	*α_s_*	*NSMS*	*k_Fxy_*	*α* *_Fxy_*
Tool	1.000										
*a*	0.145 ^a^	1.000									
*v*	−0.021	−0.017	1.000								
*m_s_*	0.008	0.002	0.012	1.000							
*s_s_*	−0.379 ^a^	0.127 ^a^	0.310 ^a^	−0.101 ^a^	1.000						
*L_s_*	−0.377 ^a^	0.149 ^a^	0.334 ^a^	−0.150 ^a^	0.863 ^a^	1.000					
*ks*	0.255 ^a^	0.120 ^a^	0.088 ^a^	0.043 ^a^	−0.093 ^a^	−0.088 ^a^	1.000				
*α_s_*	0.577 ^a^	0.201 ^a^	0.152 ^a^	0.088 ^a^	−0.311 ^a^	−0.288 ^a^	0.257 ^a^	1.000			
*NSMS*	−0.202 ^a^	0.337 ^a^	−0.130 ^a^	−0.070 ^a^	0.312 ^a^	0.322 ^a^	−0.147 ^a^	−0.011	1.000		
*k_Fxy_*	0.807 ^a^	0.092 ^a^	−0.019	0.007	−0.365 ^a^	−0.332 ^a^	0.257 ^a^	0.485 ^a^	−0.249 ^a^	1.000	
*α* *_Fxy_*	0.847 ^a^	0.111 ^a^	−0.016	0.017	−0.386 ^a^	−0.347 ^a^	0.269 ^a^	0.506 ^a^	−0.242 ^a^	0.979 ^a^	1.000

^*^ For the factor ‘Tool’, Statistica^TM^ assigned the following codes: 101—stiff tine, 102—flexible tine, 103—coulter-axis stones, 104—coulter. *a*, working depth; *v*, working speed of tool; *m_s_*, mass of stone; *s_s_*, displacement of stone; *L_s_*, specific work required to remove the stone embedded in compacted soil; *k_s_*, slope factor of the straight line for stone movement; *α_s_*, modulus of the angle for stone movement relative to the direction of the tool movement; *NSMS*, Numerical Stone Movement Scale; *k_Fxy_*, the directional coefficient of the resultant force in the horizontal plane; *α_Fxy_*, angle of the resultant force in the horizontal plane relative to the direction of the tool movement. ^a^—statistically significant correlation.

**Table 7:** Mean values and standard deviations of parameters characterizing the movement of stones embedded in the soil under the influence of contact with working elements operating at different depths and speeds.

Factor	*s_s_*	*k_s_*	*α* *_s_*	*NSMS*	*k_Fxy_*	*L_s_*
Tool						
stiff tine	0.66 ± 0.45	−0.10 ± 0.01	18.99 ± 0.30	4.00 ± 0.00	0.04 ± 0.07	327 ± 284
flexible tine	0.43 ± 0.60	0.13 ± 0.07	22.34 ± 0.43	2.49 ± 1.56	0.24 ± 0.32	125 ± 203
coulter	0.22 ± 0.14	−0.46 ± 0.21	37.13 ± 1.40	3.00 ± 0.00	0.80 ± 0.09	95 ± 58
coulter-axis stones	0.06 ± 0.06	1.33 ± 0.02	47.84 ± 0.35	5.00 ± 0.00	0.29 ± 0.29	79 ± 104
Depth *a*, m						
0.05	0.20 ± 0.51	−0.42 ± 0.05	24.02 ± 0.73	2.18 ± 1.70	0.30 ± 0.42	53 ± 138
0.07	0.45 ± 0.48	0.46 ± 0.05	27.87 ± 0.37	3.38 ± 1.03	0.33 ± 0.36	199 ± 253
0.10	0.46 ± 0.41	0.60 ± 0.02	35.59 ± 0.45	3.65 ± 0.65	0.40 ± 0.37	200 ± 193
Speed *v*, m·s^–1^						
0.83	0.24 ± 0.25	0.01 ± 0.04	24.64 ± 0.44	3.45 ± 1.12	0.35 ± 0.41	82 ± 65
1.67	0.42 ± 0.44	0.72 ± 0.07	33.41 ± 0.45	3.33 ± 1.21	0.37 ± 0.34	191 ± 239
2.22	0.61 ± 0.60	0.40 ± 0.03	31.69 ± 0.50	3.06 ± 1.13	0.33 ± 0.36	267 ± 279

*s_s_*, displacement of the stone; *k_s_*, slope factor of the straight line for stone movement; *α_s_*, modulus of the angle for stone movement relative to the direction of the tool movement; *NSMS*, Numerical Stone Movement Scale; *k_Fxy_*, the directional coefficient of the specific force, resultant in the horizontal plane; *L_s_*, specific work required to remove the stone embedded in compacted soil.

## Data Availability

Not applicable.

## Nomenclature

*a_s_*, *b_s_*, *c_s_*, and *d_s_*—thickness, width, length and equivalent diameter of the stone, respectively (mm),

*F_m_*—specific force, resultant in the axes x, y and z (N·kg^–1^);

*F_x_*, *F_y_*, *F_z_*—component forces: draught, lateral and vertical, respectively (N),

*k_Fxy_*—the directional coefficient of the specific force resultant in the horizontal plane between the *F_x_* and *F_y_* forces (–),

*k_s_*—slope factor of the straight line for stone movement (–),

*L_s_*—specific work required to remove the stone embedded in compacted soil (J·kg^–1^),

*m_s_*—mass of the stone (kg),

*NSMS*—Numerical Stone Movement Scale (–),

*s_f_*—sphericity of the stone (–),

*s_s_*—distance travelled by the stone after impact with the tool—displacement (m),

*α_Fxy_*—angle of the resultant force in the horizontal plane relative to the direction of the tool movement (°),

*α_s_*—modulus of the angle for stone movement relative to the direction of the tool movement (°),

*ρ_s_*, *ρ_sc_*—stone densities, real and computational, respectively (kg·m^–3^).
